# Complete Mesocolic Excision for Colon Cancer: Insight into Potential Mechanisms of Oncologic Benefit

**DOI:** 10.3390/cancers17162719

**Published:** 2025-08-21

**Authors:** Fotios Seretis, Antonia Panagaki, Charalambos Seretis, Maria Sotiropoulou, Michail Psarologos, Nikolaos Mamakos, Konstantinos Polyzois, Vasileios Drakopoulos, Stylianos Kapiris

**Affiliations:** 1 Third Department of General Surgery, Evaggelismos General Hospital of Athens, 10676 Athens, Greece; 2 Department of Gastroenterology, Konstantopouleio-Patision General Hospital of Athens, Nea Ionia, 14233 Athens, Greece; 3 Department of General Surgery, “Agios Andreas” General Hospital of Patras, 11527 Athens, Greece

**Keywords:** complete mesocolic excision, central vascular ligation, colon cancer, tumor deposits, lymph nodes

## Abstract

Background/Objectives: Complete mesocolic excision (CME) has recently been proposed as a radical operation for the treatment of colon cancer. Increasing evidence suggests a survival benefit from this operation, although the exact reasons for this remain largely unknown. Methods: We have undertaken a comprehensive review of the literature in PubMed and Embase databases, examining the potential mechanisms explaining this oncologic benefit. Results: Complete mesocolic excision with central vascular ligation appears to improve the rates of radial margin negativity and is associated with increased lymph node yield and improved staging for colon cancer patients by removal of apical lymph nodes and removal of skip metastasis. The en bloc removal of the cancer-related mesentery along the interfascial plane between the mesocolon and retroperitoneal structures removes en bloc tumor deposits that appear to have a significant negative effect on cancer prognosis, irrespective of lymph node status. CME is associated with decreased rates of local recurrence and improved disease-free and overall survival. The existing literature suffers from a lack of data on molecular pathology and integration of prognostic pathologic factors such as tumor deposits in patients undergoing complete mesocolic excision. Conclusions: CME confers significant benefits in terms of local control of the disease and improves cancer-specific survival. Further research on the matter is necessary to incorporate prognostic pathologic and molecular parameters.

## Simple Summary

Complete mesocolic excision with central vascular ligation appears to have oncologic benefits for colon cancer patients, with the involved mechanisms remaining elusive. When comprehensively reviewing the literature, CME was noted to secure radial margin negativity; an increased lymph node yield, including central (apical) lymph nodes; and an increased area of resected mesentery. It appears to improve colon cancer staging and prognostication, including increased detection rate of tumor deposits, which have important prognostic implications. CME-CVL appears to translate enhanced understanding of surgical anatomy into better oncologic outcomes. However, the existing literature suffers from incomplete data on molecular biology and important prognostic pathologic markers, thus failing to incorporate all current understanding of colon cancer progression and metastasis into the concept of radical surgery according to CME principles.

## 1. Introduction

Complete mesocolic excision (CME) for colon cancer was first proposed by Hohenberger et al. [1] in 2009 as a standardized procedure following the paradigm shift in rectal cancer after the implementation of total mesorectal excision introduced by Bill Heald [2]. A fundamental part of the operation is central vascular ligation, namely, proximal ligation of tumor-associated vessels from their origin, in an attempt to include the most central lymph node stations in the surgical specimen. CME entails resection of the tumor and the tumor-draining lymph nodes as a “package” bordered by embryologically defined surgical planes. The concept of complete mesocolic excision with central vascular ligation following a mesocolic plane of dissection is also reproduced in studies from Japanese centers reporting on D3 resection. A comparison of CME-CVL with D3 resection [3] yielded similar rates of involved lymph nodes, although CME-CVL seemed to be associated with an increased area of mesenteric excision, lymph node yield, and distance between the vascular tie and bowel wall.

Recent data report that CME is associated with improved lymph node yields, improved local recurrence rates, and potentially improved overall survival [4,5]. Few data provide insight, however, into the reasons for this benefit.

## 2. Materials and Methods

We have undertaken a comprehensive review of the literature in Pubmed and Embase databases with the search terms “complete mesocolic excision AND colon cancer”, “CME AND colon cancer”, “central vascular ligation AND colon cancer”, and “CVL and colon cancer”. References from retrieved papers were also manually scanned for additional articles relevant to this paper. Two independent reviewers (FS and AP) performed the literature search. A senior author (CS) resolved potential conflicts. All studies, both prospective and retrospective, reporting on patients undergoing complete mesocolic excision with central vascular ligation were included. Single case reports, studies focusing on various techniques for performing CME-CVL, and studies comparing outcomes between laparoscopic and robotic approaches were also excluded. Studies in languages other than English were also excluded. Regarding oncologic outcomes, disease-free and overall survival, as well as local recurrence rates, were used as metrics.

We have deliberately refrained from performing a systematic review of the literature with a meta-analysis, as the focus of our work has been not to demonstrate the benefit of complete mesocolic excision with central vascular dissection but rather to provide a possible explanation for these findings. Therefore, we felt that a narrative review could more effectively fulfill this purpose.

The Japanese Classification of Colorectal Carcinoma for the extent of lymph node dissection was used, with stations defined as D0 up to D3 [6]. Complete mesocolic excision with central vascular ligation corresponds to ligation of central feeding vessels and excision of draining lymph nodes at the origin of the feeding vessels, including all associated mesentery en bloc. As this was not a systematic review, we refrained from creating a flowchart for the literature search according to PRISMA (Preferred Reporting Items for Systematic reviews and Meta-Analyses) guidelines. Only papers including patients undergoing complete mesocolic excision and central vascular ligation, specifically defined, along with pathologic and/or oncologic outcomes, were included. With regard to the quality assessment of included papers, as this was a narrative rather than a systematic review of the literature, we deliberately did not use a standard tool for bias assessment as our goal was not to assess a compound outcome from included studies but rather to critically synthesize them. Papers on case reports, as well as papers in which surgical technique used was not particularly disclosed, were excluded. Articles focusing on surgical technique as well as studies focusing on comparison between open and/or laparoscopic/robotic surgery were excluded.

## 3. Results

### 3.1. Anatomic Basis of Complete Mesocolic Excision and Oncologic Implications

The concept of complete mesocolic excision arose from our revised understanding of mesentery as an “organ” and as a continuum from the duodenojejunal flexure to the anorectal junction [7]. However, the current literature lacks standardization of nomenclature [8], creating confusion among surgeons and anatomists. Insights into surgical anatomy have recently described the presence of two fasciae posterior to the intraperitoneal colon and its associated mesocolon, consisting of a visceral and parietal layer. In vivo, this two-layered fascial barrier, being avascular, permitted no lymphatic flow into the rear (retroperitoneal tissues) and was noted in vitro to halt tumor cell spread [9]. These findings formulated the basis of resection along this avascular plane of mesocolic dissection. Although various eponyms have been used to describe different areas of retrocolic fascias (namely Toldt, Fredet, Gerota, and Treitz) according to the location of interest, when using unequivoval anatomic nomenclature to describe the retrocolic fascial system, then the mesocolic plane is separated from the retrocolic plane by an interfascial double-layered fusion fascia [10]. This interfacial layer between mesocolon and parietal structures has traditionally been granted the name of the “posterior structures” from which it separates the mesocolon, namely Toldt’s fascia in the retrocolic area, space of Fredet (Fredet’s fascia) in the pre-duodenopancreatic fascia, and Gerota’s fascia along the anterior renal fascia. Interestingly enough, lymphatics have been recently described in Toldt’s fascia between the mesocolon and retroperitoneum at a distance of 0.1 mm from peritonealized mesenteric surfaces [11], thus highlighting the need for precise dissection at the interfascial plane for complete mesocolic excision. In another report from Liang et al. [12] Toldt’s fascia has been reported to be a consistent anatomical finding, while the fusion fascia of Fredet has been utilized as a crucial landmark for complete mesocolic excision with D3 lymphadenectomy in right-sided colon cancer resections [13]. Although attempts have been made at a structured description of relevant fascial and interfascial planes, exemplified by the work of Coffey et al. [14], adoption is still not universal.

The advent of laparoscopy [15] and robotics [16], with their enhanced visualization of structures under magnification, has played an important role in identification of planes by surgeons.

In an interesting single-center report from Munkedal et al. [17], when examining in the immediate post-operative period the length of residual arterial stump after right or left colectomy performed according to CME standards, the authors discovered that the length of residual arterial stamp exceeded the requirements according to CME principles, suggesting that despite efforts for the achievement of central vascular ligation, in real-life surgery, it might be of less-than-optimal standards.

### 3.2. Fascial Layers as Anatomic Landmarks and Borders

This retroperitoneal fascia has been described as a robust anatomic barrier for complete mesocolic excision in right-sided colon cancers [18]. The structural integrity of the retrocolic fascia has also been verified in studies using a combination of computed tomography and histological dissection [19].

From the surgical oncologist’s point of view, complete mesocolic excision or resection along the interfascial plane between mesocolon and retrocolic/retroperitoneal tissues has been proposed to ascertain the obtainment of a clear radial margin in patients undergoing curative resection for colon cancer. Although the obtainment of a negative radial surgical margin has been extensively described in rectal cancer surgery [20,21] as a benchmark of surgical quality and pathology-oriented outcomes with direct impact on cancer-related prognosis, this entity is less frequently encountered in colon cancers. A radial margin of less than 1 mm [22] has been associated with increased rates of recurrence, as well as decreased overall survival, irrespective of adjuvant chemotherapy status in colon cancer patients undergoing curative-intent resection. A positive radial margin has been associated with increased risk for local recurrence [23], as well as increased rates of peritoneal and liver recurrence and ultimately decreased overall survival [24]. Interestingly enough, complete mesocolic excision is associated with a decreased risk of local recurrence at 5 years compared to conventional surgery, with no effect noted on disease-free or overall survival [25]. In another report from Denmark [26], complete mesocolic excision for colon cancer significantly reduced the rates of local recurrence, with a positive effect also on distant-only and local + distal recurrence rates. These results have also been corroborated in a recent systematic review and meta-analysis including 2296 patients [27], which reported decreased rates of local recurrence with complete mesocolic excision with no effect on distant metastasis and 3-year overall survival.

### 3.3. Lymphatic Drainage of Colon Cancer

When assessing the lymphatic drainage of colonic cancer, extensive research has been undertaken in mapping techniques using indocyanine green or other tracers in an attempt to perform tumor-tailored lymphadenectomy and potentially replicate results from sentinel-lymph node excision, as is the case in breast cancer or melanoma [28]. Our increased understanding of the pathways of colonic lymph drainage has been enriched by studies on transverse colon cancer, reporting on metastatic involvement of lymph nodes in the intrapancreatic area and along the gastroepiploic arcade [29], thus raising the argument for extended lymphadenectomies at apical (D3 level) and central vascular ligation (CVL). Results from a multicenter randomized controlled trial comparing D2-level versus D3-level lymphadenectomy in colon cancer [30] reported an almost 5% rate of D3 lymph node level involvement, although no D3-level positivity without concurrent D2-level positivity was noted. A systematic review of the literature [31], despite a lack of standardization for anatomic, surgical, and pathologic terms, reported the positivity of apical lymph nodes in 1–22% for right-side tumors and up to 12% for left-side tumors. Moreover, an interesting paper from Sakamoto T et al. [32] shed further light on the role of anatomic variability of colonic vascular supply with regard to the probability of involvement of apical lymph node involvement, which might potentially explain why preoperative mapping with angio-CT colonography might improve the quality of lymph node dissection [33].

Increasing depth of tumor invasion has been associated with the risk of lymphatic metastatic spread. In a report on 348 stage I–III colon cancer patients [34], the depth of submucosal invasion (SM classification [35]) was associated with the risk of lymphatic metastasis in pericolic and intermediate lymph node stations (D1 and D2 level), as well as in the central lymph nodes (D3 level), with the authors of the study concluding that the depth of invasion beyond the SM2 level along with positive lymphovascular invasion status correlated with risk of D2-D3-level lymph node involvement, thus raising the argument for complete mesocolic excision with central vascular ligation for these tumors. When assessing only pT3 tumors, increasing tumor infiltration depth into the pericolic fat was found to be a strong prognostic factor for local and systemic recurrence for lymph-node-positive-only patients [36].

### 3.4. Tumor Deposits in Colon Cancer and Complete Mesocolic Excision

Traditionally, colon cancer surgery has been viewed as excision of the involved bowel wall with its associated lymph-node-containing mesentery in order to remove the tumor en bloc with its draining lymph node stations. However, new histoprognostic factors, such as tumor deposits, invasive tumor infiltration, and high-grade tumor-budding appear to be of significant prognostic value [37]. Unfortunately, current staging systems [38,39] for colon cancer cannot reliably discriminate cancer-related prognosis groups according to the extent of lymph node involvement while factoring in the new prognostically important players, such as tumor deposits.

Tumor deposits are focal aggregates of cancer cells in pericolic fat and mesentery, distinct from vessels, nerves, and lymphatics that appear to have a significant prognostic role [40]. In the current American Joint Commission on Cancer colon cancer staging, their presence is labeled as N1c disease. They are associated with increasing pT stage, positive perineural and lymphovascular invasion status, microsatellite tumor stability, and lymph node invasion and are independently associated with increased risk of recurrence. Interestingly enough, tumor deposit positivity is found in almost 10% of colon and rectal cancer patients and in 30–40% of those cases, no concurrent lymph node invasion is noted, according to data from 18 Surveillance, Epidemiology, and End Results (SEER) registries. In a report from the Swedish Colorectal Cancer Registry on 8146 patients [41], tumor-deposit-positive status increased the risk for local and systemic recurrence and was associated with decreased disease-free and overall survival across all N stages except for N2b patients. In an analysis from the National Cancer Database on 74,577 stage III colon cancer patients [42], patients with positive tumor deposit and negative lymph node status had equivalent 5-year overall survival with tumor-deposit-negative patients with fewer than the involved lymph nodes. Moreover, patients with both tumor-deposit- and lymph-node-positive status appeared to have the worst prognosis. Tumor deposit positivity results in a worse cancer-related prognosis across all N stages, according to a recent paper from Bhutiani N. et al. [43], with the worst prognosis being impacted by tumor deposit positivity and N2 positive lymph node status. Moreover, finding multiple tumor deposits in the resected mesentery causes a worse prognosis than finding a solitary deposit [28]. This finding suggests that the excision of a wider area of mesentery to include all potential tumor deposits might be of at least prognostic/staging significance. Interestingly enough, tumor-deposit-positive patients without lymph node metastases are less likely to receive adjuvant chemotherapy or receive fewer cycles than their lymph-node-positive counterparts, despite data demonstrating that the duration of adjuvant chemotherapy only impacts overall survival in tumor-deposit-positive patients (N1c) and not in patients with early N1 (N1a-b) status [44].

Based on those findings, performing en bloc excision of the cancer-associated mesentery should be viewed not only as removal of potentially involved lymph nodes but also of tumor deposits, despite the fact that these two entities often co-exist.

Mesentery should be viewed as an embryologically defined package that should be removed without violations of planes or injuries, as these have been pathologically confirmed to disrupt lymph nodes and vessels, as reported in an excellent paper by Kataoka et al. [45], causing “iatrogenic tumor deposits”.

Unfortunately, existing studies on complete mesocolic excision for colon cancer have only focused on the extent of lymphanectomy and its potential oncologic benefits across the various stages of the disease. However, according to a recent report [46], tumor-deposit-positive patients without lymph node metastasis fare worse than patients without deposits but with up to 1–3 involved lymph nodes. In the same study, tumor-deposit-positive patients appear to have the same prognosis as patients without tumor deposits but with equal to or more than four involved lymph nodes. Therefore, it may be possible that failing to take into account tumor deposits when considering the benefit of complete mesocolic excision might act as a confounding factor.

### 3.5. Benefit of Extended Lymphadenectomy in Colon Cancer

Colon cancer progression involves hematogenous and lymphatic spread to locoregional lymph nodes, distant lymph nodes, and metastatic sites in other organs, such as the liver. Although the mechanisms of cancer progression and metastasis remain largely unknown, two main dogmas have dominated our understanding of these complex processes, namely the Halsted and Fisher models [47]. On one hand, the Halsted model assumes a stepwise progression in an orderly fashion from the primary tumor into tumor-draining lymph nodes and from thereon into the systemic circulation, thus assuming lymphogenous spread as a precursor to hematogenous spread. The Halsted model has dominated surgical oncology thinking for many years and has been the keystone for performing lymphadenectomy in the tumor-draining lymph node basins. On the other hand, the Fisher model assumes that the release of metastatic cells is an early and random event. According to the Fisher hypothesis, performing surgery and extended lymphadenectomies in central lymph nodes (D3 level) might not be of benefit, as their involvement is considered a late event in cancer progression, and thus no benefit is to be derived from their removal. Our increasing understanding of the impact of tumor deposits in both local recurrence and risk for systemic spread might be the link between those two theories, providing a possible explanation as to why a local phenomenon of tumor spread into the nearby mesentery might actually confer a risk for systemic metastasis irrespective of lymph node involvement and might confer a risk for local recurrence if the visceral–embryologically defined bowel mesentery package is violated by resection deviant from interfascial surgical planes. To complicate matters even more, tumor clonal heterogeneity has been demonstrated among the primary tumor versus involved lymph nodes and solid-organ metastatic signs and has been described to vary in left versus right-sided tumors [48].

When assessing the oncologic benefit of complete mesocolic excision (CME) with central vascular ligation (CVL) in colon cancer, CME-CVL has been demonstrated to confer a significant benefit in disease-free survival across stages I–III of disease [49]. Corroborating data on 446 patients with stage III colon cancer have also demonstrated a survival benefit for CVL even for patients with involvement of D3 level lymph nodes [50]. In a direct comparison of CME versus non-CME-plane resection for colon cancer [51], CME has been reported to confer a significant benefit in the obtainment of negative circumferential and R0 resection margins, translating into improved 5-year overall survival rates, especially in patients with stage II disease and in stage III patients with no apical lymph node involvement. In another report comparing CME to non-CME colon resections, CME was reported to also have a significant benefit in local recurrence-free survival [52]. When focusing solely on patients with stage I-II disease, CME has also been reported to confer a significant benefit in disease-free and cancer-specific survival when compared to non-CME surgery [53]. In an interesting report from Galizia et al. [54], CME surgery was associated with increased lymph node yields and, more interestingly, increased detection of tumor deposits, thus leading to upstaging and delivery of adjuvant chemotherapy in a significant number of patients, with the authors also reporting no locoregional recurrencies in their cohort.

Also of clinical importance is the fact that the rate of skip metastasis as assessed in patients undergoing CME-CVL has been reported to be up to 19.8% [55], with the authors of this study reporting that securing an adequate lymph node harvest including apical lymph nodes by the combination of CME-CVL led to upstaging of the disease in 4.5% of patients, thus being critical for proper staging.

## 4. Discussion

CME-CVL has been proposed following the treatment paradigm of rectal cancer, where TME changed the landscape and provided evidence that resection along the embryologic planes conferred a significant reduction in local recurrence rates but also a significant cancer-specific survival advantage. Resection along the interface between mesocolic planes and the retroperitoneum enables the removal of tumor and tumor-associated mesentery en bloc in an intact fascial envelope, thus precluding tumor cells and/or involved lymph nodes to be left intact. Moreover, central ligation of feeding vessels ensures removal of apical lymph nodes, as well as an adequate length of mesentery, as exemplified by the length between the bowel wall and vascular tie. This approach has been demonstrated to confer decreased local recurrence rates attributable to improved rates of radial margin involvement and R0 resection rates. Moreover, extensive resection of tumor-associated mesentery also encompasses potential tumor deposits that have been described to increase the risk for both local and systemic failure. However, the literature examining the rate of detection of tumor deposits in patients undergoing CME-CVL is scarce, especially in patients with no lymph node involvement.

From an oncologic point of view, the CME-CVL approach is associated with increased lymph node yields in surgical specimens, thus enabling accurate staging. We have found that a significant proportion of patients are upstaged either by increased detection rates of tumor deposits or by adequate lymph node sampling, including apical (corresponding to D3 level) stations. A consistent survival benefit was noted in studies incorporating patients with stages I-III of disease. Therefore, complete mesocolic excision should firstly be viewed as an accurate staging procedure, thus leading patients at increased risk for disease failure to receive adjuvant treatments that would otherwise not have been offered to them. Most importantly, though, CME-CVL should be viewed as an extensive resection performed with curative intent, as it improves local control of disease and confers a significant survival benefit to patients. In our opinion, the dictum of Halsted’s hypothesis of tumor spread and metastasis has affected our perception of CME-CVL as an extended lymphadenectomy procedure, although evidence suggests that extensive lymphadenectomies primarily affect local and not systemic recurrence rates. When considering the Fisher hypothesis of colon cancer spread and metastasis, CME-CVL has the potential to radically remove tumor clones that have increased metastatic potential, namely tumor deposits and involved lymphatic stations at the D2 and D3 levels. This concept derives from the very definition of tumor deposits as deposits within the tumor-associated mesentery, distinct from lymph nodes and vessels. Therefore, the radicality of mesenteric excision entailed in complete mesocolic excision and of all draining lymph nodes, including central stations, by default ensures removal of potential tumor deposits. What is also known is the fact that tumor deposit positivity rates increase as the N stage increases and are evident even in early-stage tumors [56,57]. It might be possible, therefore, that the benefit of extended lymphadenectomy with CME-CVL could be attributed to the excision of tumor deposits along with the extended lymphadenectomy, with N status essentially being a confounding factor. This hypothesis, in our opinion, might bring new insights into the perception of colon cancer oncologic resections, especially from the CME-CVL point of view.

**Figure 1:**
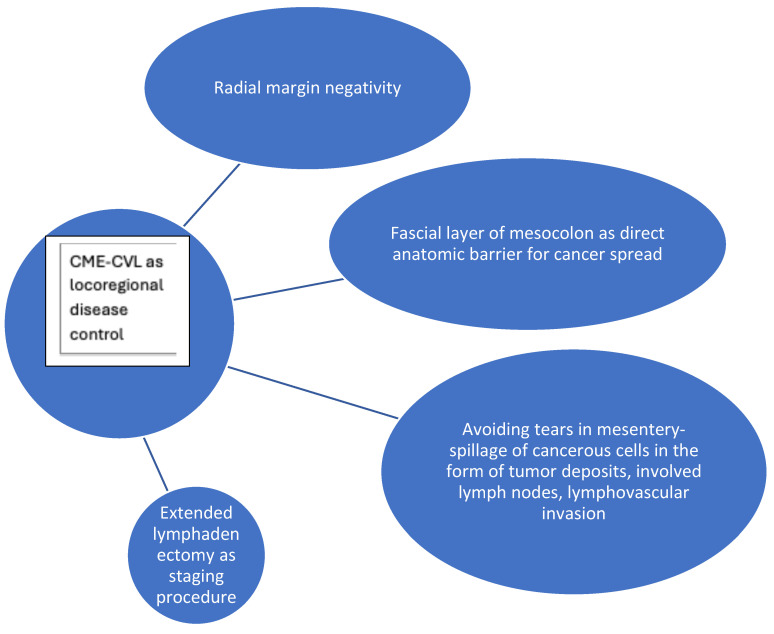
Conceptual diagram for mechanisms of CME-CVL for locoregional disease control of colon cancer.

**Figure 2:**
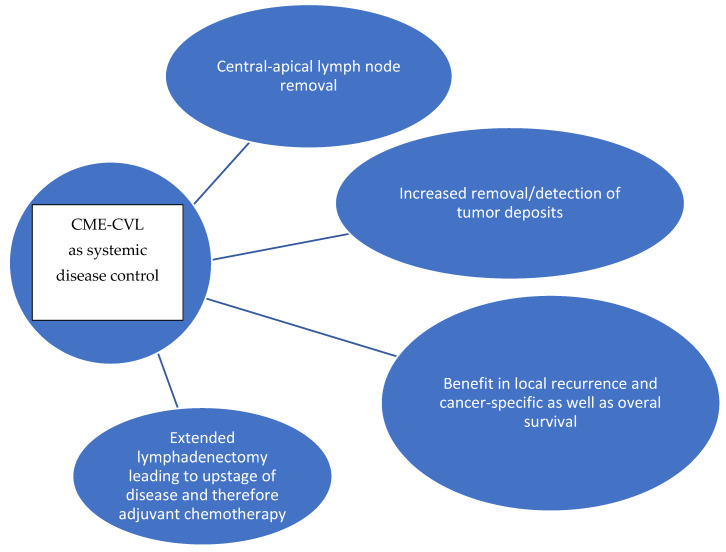
Conceptual diagram for mechanisms of CME-CVL for systemic disease control of colon cancer.

The aforementioned concepts are graphically summarized in Figure 1 and Figure 2, depicting the potential mechanisms with which CME-CVL might enhance locoregional and systemic control of colon cancer, respectively.

Our work also suffers from limitations. Although early studies reporting on surgical specimen quality [58,59,60,61] did demonstrate increased area of mesenteric resection and increased lymph node yields, subsequent studies reporting on oncologic outcomes of CME-CVL have not reported on quality control of surgical specimens, thus leading to presumptions only that proper-quality CME surgery has been performed. It is interesting to note that a multicenter study of complete mesocolic excision for colon cancer with centralized expert pathologic assessment of the quality of surgical specimens, where proper CME was almost universally performed, reported only on a possible benefit for stage III disease with regard to overall survival but not disease-free survival [62]. In our opinion, these findings might challenge the concept that CME-CVL is associated with a benefit consistently across all cancer stages; thus, a more elaborate approach might be needed with more precise patient selection. However, at the present time, this remains to be elucidated until more data become available. The lack of standardized quality control of surgical specimens creates confusion, potentially with regard to the exact benefit of CME-CVL, as a lack of observed benefit in some studies might be a mere reflection of “poor-moderate quality” surgical technique. Conversely, the benefit of CME-CVL might be a reflection of proper oncologic technique in comparison to standard approaches. Therefore, comparisons should ideally be performed between the two techniques when the proper technique with standardized pathologic quality control is employed. In the most recently published systematic review and meta-analysis on CME for colon cancer, although a benefit has been demonstrated for disease-free and overall survival [4], the authors have reported that the quality of evidence is low and is based on studies at risk of significant bias. For these reasons, we believe that CME-CVL results should be meticulously studied, considering the maintenance of surgical standards and pathologic reporting. There is a low quality of evidence and a significant risk of bias in the included studies; therefore, the strength of conclusions regarding CME-CVL might be undermined, and potential benefits might be overestimated. Moreover, the existing literature on CME-CVL suffers from the lack of data on the molecular profile of colonic tumors; thus, tumor biology is not taken into account. Further insight into the molecular profiling of tumors undergoing CME, with a focus especially on microsatellite stability status, might be the key to understanding which patients might benefit the most from such an extensive resection, thus allowing a tailored approach. A lack of molecular data might potentially group together patients with different biologic behavior and distinct prognoses. Moreover, no data exist to our knowledge on the outcomes of patients undergoing neoadjuvant chemotherapy for colon cancer and then undergoing curative intent surgery [63] from the complete mesocolic excision point of view. This is of particular importance, as an increasing number of patients are undergoing neoadjuvant treatment, according to modern oncologic outcomes. Neoadjuvant treatment might have an effect on lymph node yield after resection, as well as their positivity rate, so the benefit of extended lymphadenectomy in the case of CME-CVL might be less clear. Finally, we have highlighted the scarcity of pathologic data in the CME-CVL literature on tumor deposits, tumor budding, and other important pathologic prognostic factors. A lack of data on key prognostic factors for colorectal cancer, such as tumor budding or tumor deposits, might act as a significant confounding factor. Grouping together patients with low and high tumor budding scores and/or patients with and without tumor deposits is essentially grouping together groups at a lower and higher risk for cancer progression and metastasis, so applying CME-CVL in this setting might either fail to demonstrate the magnitude of the benefit of this technique, or on the other hand might falsely demonstrate better outcomes in the low-risk (low tumor budding, absent tumor deposits) groups. In our opinion, further research into this field is required.

## 5. Conclusions

Complete mesocolic excision for colon cancer is a revolutionary concept with a direct positive impact on local recurrence rates and survival benefits for the patients. Potential mechanisms for this effect appear to be the en bloc excision along embryologically defined planes of tumors and its associated mesentery with tumor-containing lymph nodes and tumor deposits, the increased lymph node yield, the excision of central (apical) lymph nodes, and the proper staging, including a significant proportion of patients with upstaging and potentially further adjuvant treatments. The existing literature suffers from significant limitations that prevent a unifying approach to the existing literature on the radical surgical treatment of colon cancer and its oncologic implications. Further studies are needed with careful control of surgical and pathologic quality assessment, as well as inclusion of relevant molecular biomarkers, so that CME-CVL is put into the current context of surgical oncology for colonic malignancies.

## Abbreviations

The following abbreviations are used in this manuscript:

CME	Complete mesocolic excision
CVL	Central vascular ligation
TME	Total mesorectal excision
